# Cardiac Rehabilitation After Left Ventricular Assist Device Implantation: A Narrative Review

**DOI:** 10.3390/jcm15031089

**Published:** 2026-01-30

**Authors:** Rita Gravino, Luigi Falco, Dario Catapano, Cristiano Amarelli, Fabio Valente, Marina Verrengia, Claudio Marra, Emilio Di Lorenzo, Pierino Di Silverio, Michelle Kittleson, Daniele Masarone

**Affiliations:** 1 Department of Cardiology, AORN dei Colli Monaldi Hospital, 80131 Naples, Italyluigifalco94@libero.it (L.F.); emilio.dilorenzo@ospedalideicolli.it (E.D.L.); 2 Department of Cardiac Surgery and Transplant, AORN dei Colli Monaldi Hospital, 80131 Naples, Italy; cristiano.amarelli@ospedalideicolli.it (C.A.);; 3 Campania Region Transplant Center, AORN dei Colli Monaldi Hospital, 80131 Naples, Italy; 4 Department of Cardiology, Smidt Heart Institute, Cedars-Sinai Medical Center, Los Angeles, CA 90048, USA

**Keywords:** cardiac rehabilitation, left ventricular assist device, advanced heart failure, exercise-based rehabilitation

## Abstract

Over the past decade, given safety, reduced heart failure-related hospitalizations, and, above all, 5-year mortality rates nearly identical to those of heart transplants, left ventricular assist devices (LVADs) have increasingly become a treatment option for patients with advanced heart failure. However, improvements in functional capacity after LVAD implantation are minimal or modest, depending on pre-implantation right ventricular function, the patient’s hemodynamic status, the optimization of guideline-directed medical therapy, and noncardiac factors (physical deconditioning, skeletal muscle alterations, anemia, and alterations in alveolar gas exchange). Therefore, cardiac rehabilitation (CR) is a fundamental element from the early stages after LVAD implantation, as it is not only safe but also highly effective, leading to improved functional capacity and fewer episodes of worsening heart failure, and may be associated with reduced mortality. To perform safe and effective CR in patients with LVADs, it is crucial to account for the unique issues in this group. This includes the difficulty of detecting an arterial pulse with standard tools during CPR and the importance of closely monitoring the transmission line and LVAD controller to prevent unintended damage. Overall, the clinical trial indicates that exercise-based CR has the potential to improve functional capacity. Furthermore, some data suggest that CR is associated with fewer HF-related hospitalizations and may be linked to lower mortality; however, there is no consensus on this matter, partly because most studies supporting this assertion are observational.

## 1. Introduction

The left ventricular assist device (LVAD) has become a crucial treatment option for patients with advanced heart failure (advHF), offering a 5-year survival rate after implantation that is nearly comparable to that of transplantation. It serves as an alternative or bridge to a heart transplant [1]. Data from the 16th Annual Report of the Society of Thoracic Surgeons (STS) Interagency Registry for Mechanically Assisted Circulatory Support (INTERMACS) between 2015 and 2024 show that 28,029 continuous-flow implants were performed during this period [2]. This upward trend is driven by technological advancements, increased awareness of LVAD options, and broader indications that now include patients with less severe heart failure.

Cardiac rehabilitation (CR) plays a crucial role in recovery following cardiac surgery or acute cardiac events, including worsening heart failure (HF) [3]; however, despite guidelines from scientific societies, referrals of LVAD patients to CR remain insufficient, and there is limited evidence regarding the feasibility and benefits of early post-acute CR in this population. Given the conflicting data regarding certain aspects of CR in LVAD recipients, this narrative review will critically examine the existing literature on the role of CR following LVAD implantation. For this purpose, a comprehensive literature search was conducted utilizing PubMed, SCOPUS, ScienceDirect, and PEDro databases, employing the keywords “left ventricular assist device OR LVAD OR HeartMate III AND cardiac rehabilitation OR exercise OR physical training.” All studies on CR in adult patients with LVAD implants, including both early and late rehabilitation phases, were included.

## 2. Physiology and Pathophysiology of LVAD at Rest and During Exercise

Due to better survival rates, fewer complications, and longer device lifespan, pulsatile flow LVADs have been entirely replaced by continuous flow LVADs [4]. Today, HeartMate III is the sole continuous flow (centrifugal) device used in Western countries. At rest, the cardiac output and cardiac index of a patient with a HeartMate III are determined by the combination of LVAD flow and the aortic valve opening frequency [5]. The pressure difference between the inflow and outflow cannulas (representing mean arterial pressure) determines the flow through the HeartMate III. Right ventricular function also influences left ventricular preload and overall cardiac output; thus, in patients with right ventricular dysfunction after LVAD implantation, cardiac output can be reduced at rest even if the mentioned variables are normal [6].

### 2.1. Determinant of Functional Capacity in LVAD Recipients

The functional capacity of LVAD recipients mainly depends on right ventricular function, the device’s sensitivity to afterload, chronotropic competence, and native myocardial contractility. However, as shown in Table 1, other extracardiac factors, such as anemia, physical deconditioning, exercise-induced hypertension, and changes in alveolar gas exchange, also affect the functional capacity of an LVAD recipient [7]. At a set LVAD rate, maintaining euvolemia and achieving a cardiac output above 4–5 L per minute are sufficient to significantly reduce mortality, reduce the frequency of worsening HF episodes, and improve quality of life, but not to guarantee normal hemodynamics during exercise. Evidence shows that LVAD patients’ exercise hemodynamics resemble those with heart failure with reduced ejection fraction, indicating LVAD support maintains normal hemodynamics only at rest, not during activity [8].

In a study involving 16 LVAD recipients that evaluated the response of cardiac and pump parameters to maximal or submaximal exercise, it was demonstrated that aortic valve opening frequency and increased heart rate—parameters correlated with residual left ventricular contractility—rather than changes in pump flow, were associated with oxygen uptake at peak exercise (pVO_2_) [9]. These findings differ from a study involving 14 patients with HeartMate II, which documented that cardiopulmonary exercise testing with incremental LVAD pump speed every 2 min significantly enhanced maximum aerobic capacity compared to a fixed pump speed (pVO_2_ 15.4 ± 5.9 mL/kg/min vs. 14.1 ± 6.3 mL/kg/min; *p* =0.012). This represented a net increase of 9.2% in pVO_2_. Additionally, ventilatory efficiency metrics improved in the group with increased LVAD pump speed, likely due to better left ventricular unloading during exercise [10]. Moreover, Camboni et al. observed an exercise-induced increase in mean pulmonary artery pressure (16 ± 2.4 vs. 27 ± 2.8 mmHg, *p* < 0.001), pulmonary artery wedge pressure (9 ± 3.3 vs. 17 ± 5.3 mmHg, *p* = 0.01), and cardiac output (4.7 ± 0.5 vs. 6.2 ± 1.0 L/min, *p* = 0.008) in 8 patients supported at fixed speeds that permit aortic valve opening and maintain pulsatility. These data clearly show that increasing the frequency of aortic valve opening does not improve exercise capacity in LVAD recipients [11]. 

Conversely, residual native left ventricular contractility seems to be the key factor influencing exercise capacity. In a study involving 30 patients with HeartMate II, Noor et al. showed that in the entire study population, pVO_2_ was positively correlated with left ventricular ejection fraction (LVEF) at both a pump speed of 9000 rpm (r = 0.41, *p* = 0.03) and a pump speed of 6000 rpm (r = 0.50, *p* = 0.01). Furthermore, when the population was divided according to ejection fraction values, it was noted that the response to changes in pump speed differed between the two groups. In fact, in patients with higher LVEF (>40%), the impact of LVAD pump speed reduction (from 9000 rpm to 6000 rpm) was minimal [pVO_2_ 21.4 ± 4.8 mL/kg/min vs. 20.8 ± 5.5 mL/kg/min, *p* = 0.38]. Conversely, in the lower LVEF group (<40%), pVO_2_ was lower at both speeds; [pVO_2_ 17.2 ± 5.3 vs. 14.7 ± 5.9 mL/kg/min, *p* = 0.02]. Finally, patients in the lower LVEF group also showed ventilatory inefficiency—documented by a higher VE/VCO2 slope value—that increased further with speed reduction (38.4 ± 5.3 vs. 47.1 ± 16.2; *p* = 0.009) [12]. Confirming this, a study that evaluated invasive haemodynamics and maximum exercise capacity in 45 patients supported by axial (n = 12) and centrifugal (n = 33) LVAD used a multivariate model that showed the parameters most predictive of pVO_2_ were cardiac output assessed using the Fick technique and peak arteriovenous oxygen difference [13]. This study, therefore, confirmed that the main parameter influencing exercise capacity is residual native left ventricular contractility, as demonstrated by an increase in Fick cardiac output with minimal contribution from LVAD pump flow.

### 2.2. Effect of LVAD Implant on Functional Capacity

The impact of LVAD implantation on pVO_2_ remains a subject of ongoing debate, with some studies indicating a neutral effect and others demonstrating an improvement. Notably, a study involving 25 patients who underwent Heart Mate II implantation [14] showed that the cardiopulmonary test conducted twelve months post-implantation revealed a modest increase in peak VO_2_ (11.5 vs. 12.4, *p* = 0.17). Additionally, a significant improvement in gas exchange during exercise was observed, evidenced by a reduction in VE/VCO_2_ (39.4 vs. 35.7; *p* = 0.017. Similarly, Felix et al. demonstrated that, in a study involving 105 patients who received HeartMate II (75 patients), HeartMate III (8 patients), and HeartWare (22 patients) implants, there were improvements in maximal workload and peak oxygen uptake observed at 1-year post-LVAD implantation, based on serial CPET assessments at 6 and 12 months. One year after LVAD implantation, patients experienced an increase in pVO_2_ (16.5 ± 5.0 to 17.2 ± 5.5 mL/kg/min; *p* = 0.008), representing a 7% absolute growth. There was also a rise in the anaerobic threshold (10.8 ± 3.1 to 12.1 ± 3.7 mL/kg/min; *p* ≤ 0.001). Additionally, the respiratory exchange ratio was significantly lower at 12 months (1.21 ± 0.11 to 1.18 ± 0.11; *p* = 0.021), suggesting decreased anaerobic metabolism or effort. In multivariable linear regression analysis, key predictors of increased pVO_2_ included younger age, lower body mass index, lower maximal workload, and higher anaerobic threshold during the cardiopulmonary exercise test at 6 months [15].

### 2.3. Role of the Right Ventricle

Despite the well-established prognostic significance of right ventricular dysfunction in LVAD patients and the understanding that both early and late right heart failure post-LVAD can limit exercise capacity, studies assessing its impact on functional capacity have shown mixed results. For instance, Jung et al. [10] found no correlation between tricuspid annulus plane systolic excursion (TAPSE) or right ventricular diastolic diameter with baseline pVO_2_, nor did these measures relate to responses to increasing pump speed. On the other hand, a study involving 83 LVAD patients tested at CPET 7 months after implantation found that the severity of tricuspid regurgitation, a proxy for right ventricular function after LVAD, was linked to reduced pVO_2_ [16]. Supporting this, a study of 22 LVAD patients demonstrated that right ventricular systolic function played a key role in elevating VO_2_p during exercise in response to increased CF-LVAD speeds. Specifically, a TAPSE value > 13 mm was a significant predictor of a ΔpVO_2_ ≥ 3 in logistic regression analysis (*p* = 0.05) [17]. 

In summary, the functional capacity and quality of life for LVAD recipients are impacted by various factors. Many of these factors can be influenced and improved through targeted early- and late-stage CR protocols, underscoring the importance of CR in effective patient management.

## 3. CR in Patients with HF: General Principles

The World Health Organization (WHO) defines cardiac rehabilitation (CR) as activities aimed at addressing the disease’s root cause and optimizing physical, mental, and social well-being, enabling patients to regain or maintain community participation [18]. For heart failure (HF) patients, international guidelines recommend that CR programs focus on developing long-term management skills [18]. Goals include improving exercise capacity, quality of life, medication adherence, device management, and reducing hospitalizations and mortality [19]. Over three decades, CR has expanded from solely physical exercise to multidisciplinary protocols that include assessment, education, lifestyle modification, psychological support, and barrier reduction. Success depends on collaboration among patients, caregivers, and providers, with guidelines that emphasize a multidisciplinary team approach. Patients are initially assessed and referred by a cardiologist; the team typically includes specialized nurses, physiotherapists, and dietitians, and may also include psychologists, social workers, and pharmacists [20,21]. Recent meta-analyses show CR adds benefits beyond drug therapy, with many experts considering it the fifth pillar of HFrEF treatment. The 2022 Cochrane review of 60 studies involving 8728 HFrEF patients found that CR reduces hospitalizations by 25–30% and improves quality of life, oxygen extraction, and maximum oxygen consumption, with benefits consistent across HF severity [22]. The ExTraMATCH II meta-analysis of 3990 patients confirmed reductions in hospital readmissions and improvements in quality of life, regardless of demographic or clinical factors [23]. Therefore, guidelines recommend offering exercise-based CR to all HFrEF patients. Given that conducting CR in patients with LVADs necessitates the consideration of certain aspects unique to this population (such as the absence of a detectable arterial pulse using standard instruments during CR and the critical importance of meticulous attention to the transmission line and LVAD controller), the foundational principles, benefits, and practical considerations of CR for patients with LVADs are delineated below.

## 4. CR Post LVAD Implant: Rationale and Results

Over the past decade, the growing number of LVAD recipients accessing CR programmes has created an unprecedented challenge for healthcare professionals involved in these programmes, particularly those based on physical exercise. Nevertheless, all patients with an implanted LVAD should participate in exercise-based CR, given the substantial benefits these programmes offer. 

**Figure 1:**
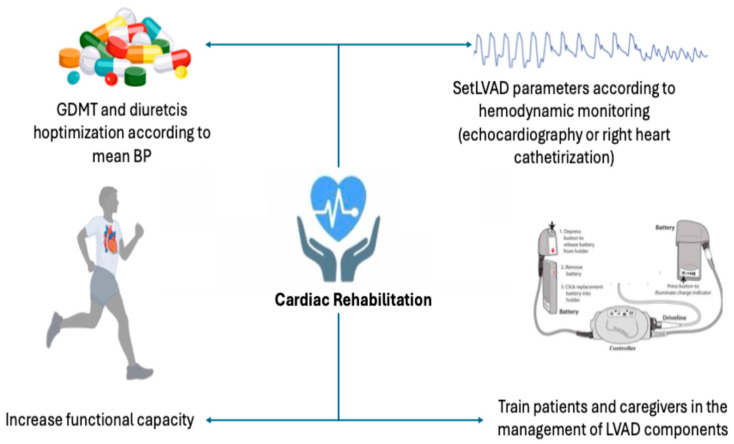
Main goals of cardiac rehabilitation. GDMT: guidelines directed medical therapy, BP: blood pressure, LVAD: left ventricular assist device.

CR after LVAD implantation is divided into early and late phases. Both phases focus on enhancing the patient’s mobility and quality of life, optimizing guideline-directed medical therapy, and developing skills in managing LVAD equipment (Figure 1). They also include caregiver training and cognitive strengthening.

Throughout both phases, it is essential to regularly optimize guideline-recommended medical and diuretic therapies to manage residual HF symptoms. Additionally, adjustments to LVAD parameters should be based on echocardiographic data or right heart catheterization measurements [24]. Early inpatient rehabilitation is frequently required owing to physical deconditioning, extended bedrest, and patient frailty [25]. The primary aim is to enhance the Functional Independence Measure (FIM) scale, which evaluates the independence level of hospitalized patients across six domains: self-care, transfers, locomotion, sphincter control, communication, and social cognition [26]. A study of 19 patients showed that inpatient CR significantly improves the FIM score (mean admission score 62.1 ± 10.9 vs. mean discharge score 89.7 ± 10.9, *p* < 0.001) [27]. Late CR, which begins after hospital discharge, primarily aims to improve functional capacity and quality of life [28]. In fact, exercise capacity after LVAD implantation is generally limited, with activity restricted to low-intensity workloads, reflecting compromised maximum oxygen consumption similar to that seen in patients with HFrEF. CR, particularly exercise-based CR, significantly improves these parameters [29].

LVAD alone can enhance haemodynamic conditions, thereby boosting a patient’s functional capacity. In a pilot study involving eight patients with advanced HF who received LVADs and underwent serial cardiopulmonary exercise testing (CPET), significant improvements were observed in maximum pVO_2_ (12.6 ± 2.0 to 14.9 ± 1.6 mL/kg/min; *p* < 0.01) and AT (7.8 ± 1.7 to 10.9 ± 1.7 mL/kg/min) after six months [30]. However, CR results in a notable increase in functional capacity, surpassing the advantage of LVAD implantation alone, as conclusively demonstrated by the Rehab-VAD randomised controlled trial. In this trial, 26 patients receiving LVAD implantation were randomly assigned in a 2:1 ratio to either CR (18 sessions over six weeks) or usual care. The study demonstrated that patients who underwent CR exhibited improved VO_2_ max values (13.6 ± 3.3 vs. 15.3 ± 4.4 in the CR group and 11.2 ± 2.0 vs. 11.8 ± 2.0 in the usual care group; *p* = 0.222) and a higher anaerobic threshold compared to those in the usual care group (10.0 ± 2.1 vs. 10.9 ± 2.1 in the CR group and 9.1 ± 0.7 vs. 9.3 ± 1.0 in the usual care group; *p* = 0.315). Furthermore, patients who completed CR demonstrated a significant improvement in quality of life, with an average increase of 14.4 points on the Kansas City Cardiomyopathy Questionnaire, compared with a mere 0.5-point increase in the usual care group. Item-by-item analysis revealed that this improvement was associated with improvements in physical limitations (*p* = 0.002), symptom frequency (*p* = 0.013), symptom burden (*p* = 0.042), overall quality of life (*p* = 0.002), and social limitations (*p* = 0.021) [31]. Finally, patients with LVADs who improve their functional capacity CR tend to have better long-term survival. A recent multicenter retrospective study found that patients actively engaged in CR programs had a 96% one-year survival rate (95% CI, 91–98). In contrast, those unable to participate in CR had a higher risk of death (hazard ratio, 2.85; 95% CI, 1.49–5.44; *p* < 0.01) and more hospitalizations (incidence rate ratio, 1.74; 95% CI, 1.14–2.66; *p* = 0.01) [32]. In addition to enhancing cardiopulmonary test performance, cardiac rehabilitation also increases distance covered during the six-minute walk test (6MWDT). In the Rehab study, CR significantly increased distance covered in the 6MWDT by 52.3 metres compared with the control group. In another study, Schmidt et al. [33] employed a muscle-strength rehabilitation protocol spanning 3 to 5 weeks, conducted 5 to 7 days per week, involving three sets of 20 repetitions. This protocol incorporated lower limb training and ergometric cycling. Notable improvements were observed in distance covered during the 6MWT, increasing from 325 ± 106 m to 405 ± 77 m (*p* < 0.001). Ultimately, the analysis of the clinical trial (Table 2) indicates that exercise-based CR has the potential to improve functional capacity (as measured by pVO_2_ or distance covered at the 6MWDT). Furthermore, some data suggest that CR is associated with fewer HF-related hospitalizations and may be linked to lower mortality; however, there is no consensus on this matter, partly because most studies supporting this assertion are observational.

## 5. Practical Aspect of CR After LVAD Implantation

CR in patients with LVAD necessitates a multidisciplinary approach, with close cooperation between the advanced HF specialist and the CR specialist. This teamwork should address all elements of the CR program, including safety concerns (such as clinical history, risk evaluations, exercise hazards, cognitive or psychological issues), related factors (like professional and recreational needs, orthopedic limitations, premorbid condition, current activities, health and fitness goals), and non-cardiac issues. Practical guidance will now be provided to help implement CR for LVAD patients, aiming to support all centers managing LVAD recipients. Since well begun is half done, the most important thing is that CR must be started after the patient’s haemodynamic stability is achieved (LVAD flow > 3 L/min, mean arterial pressure > 70 mmHg, oxygen saturation > 90%, no chest pain, dyspnea at rest, or signs of worsening heart failure) [36]. In patients with LVADs, cardiac output relies heavily on preload [37]; therefore, physical activity should be confined to the anaerobic threshold, since excessive peripheral vasodilation beyond this limit can lead to symptomatic hypotension, especially when the native heart’s contribution is limited. Likewise, during CR, maintaining proper volume status is essential to avoid flow reductions and “zero flow” alarms caused by flow drops or aspiration of the inflow cannula [38]. Volume status may be assessed via echocardiography before and after each CR session, and fluids may be administered if needed. Since, especially in the immediate post-operative period, patients with LVADs may experience autonomic dysfunction [39], which can cause episodes of symptomatic hypotension, it is important to be aware of this to prevent or quickly manage such episodes during CR. Finally, it is essential to handle the LVAD driveline and controller with extreme care, as any damage can have catastrophic consequences for the patient [40].

## 6. Conclusions

Advances in LVAD technology have improved survival rates, enhanced quality of life, and reduced adverse events related to haemocompatibility in LVAD recipients. The subsequent challenge is to increase functional capacity and reduce hospital admissions for heart failure. In this context, initiating inpatient CR and continuing it after discharge is crucial for improving patients’ functional capacity with LVADs. Although current experience and evidence regarding CR in LVAD patients are limited, it is evident that this approach can improve key functional capacity metrics in cardiopulmonary exercise testing (notably VO_2_p and anaerobic threshold), increase the distance covered at 6MWDT, enhance patients’ quality of life and psychological well-being (particularly by alleviating depression symptoms), decrease hospital readmissions, and may be associated with reduced mortality. Consequently, CR programs for LVAD patients should be established in all centers treating this specific group of advanced heart failure patients. These programs should be delivered by skilled, trained professionals and based on a multidisciplinary assessment that covers psychological health, risk factor management, social support, caregiver training, and physical therapy. Future well-designed randomized clinical trials will be necessary to identify the most effective CR protocol in LVAD recipients.

## Figures and Tables

**Table 1:** Cardiac and extracardiac factors influencing functional capacity in patients with LVADs.

**Cardiac and LVAD Factors**
Chronotropic incompetence
Right ventricular function and pulmonary pressure
Native left ventricular contractility
Native heart valvular diseases
LVAD sensitivity to afterload
**Extracardiac factors**
Anemia
Skeletal muscle abnormalities
Alteration in alveolar gas exchange
Hypertension induced by physical exercise
Physical deconditioning

**Table 2:** Summary of the main trial of CR in LVAD recipients.

Study	Numbers of Patients	Intervention Duration (Weeks)	Main Outcome
Marko [29]	41	5	Improvement pVO_2_
Kerrigan [31]	26	6	Improvement pVO_2_
Schmidt [33]	10	3	Muscular strength in all trained muscles
Scaglione [34]	25	4	Improvement pVO_2_
Alvarez Villela [35]	12	5	Improvement pVO_2_

## Data Availability

No new data were created or analyzed in this study.
